# Exposure to Toxic Heavy Metals Can Influence Homocysteine Metabolism?

**DOI:** 10.3390/antiox9010030

**Published:** 2019-12-28

**Authors:** Caterina Ledda, Emanuele Cannizzaro, Piero Lovreglio, Ermanno Vitale, Angela Stufano, Angelo Montana, Giovanni Li Volti, Venerando Rapisarda

**Affiliations:** 1 Department of Clinical and Experimental Medicine, University of Catania, 95123 Catania, Italy; ermannovitale@gmail.com (E.V.); vrapisarda@unict.it (V.R.); 2 Department of Health Promotion Sciences Maternal and Infantile Care, Internal Medicine and Medical Specialities “Giuseppe D’Alessandro”, University of Palermo, 90127 Palermo, Italy; emanuele.cannizzaro@unipa.it; 3 Interdisciplinary Department of Medicine, University of Bari “Aldo Moro”, 70124 Bari, Italy; piero.lovreglio@uniba.it (P.L.); stufano.a@gmail.com (A.S.); 4 Department of Medical Science, Surgical Science and advanced Technologies “G.F, Ingrassia”, University of Catania, 95123 Catania, Italy; angelomontana49@gmail.com; 5 Department of Biomedical and Biotechnological Sciences, University of Catania, 95123 Catania, Italy; livolti@unict.it

**Keywords:** methionine, MTHFR, vitamin B_6_, vitamin B_12_, folate, lead, chromium, cadmium, mercury

## Abstract

Background: Homocysteine is a sulfur amino acid whose metabolism is activated in two pathways: remethylation to methionine, which requires folate and vitamin B_12_, and transsulfuration to cystathionine, which needs pyridoxal-5’-phosphate. High homocysteine level increases the risk of developing heart disease, stroke, peripheral vascular diseases, and cognitive impairment. Some evidence showed that exposure to these metals increased plasma homocysteine levels. Methods: A systematic review was carried out to clarify the relationship between homocysteine blood levels and exposure to toxic heavy metals (Lead, Cadmium, Mercury, and Chromium). Results: The results of this systematic review indicate that exposure to Pb, Cr, Cd, and Hg is connected with nonphysiological homocysteine levels or vitamin B_12_ and folate serum concentrations. Conclusions: These findings reinforce the importance of involvement in exposure to heavy metals in homocysteine metabolism. This supports the role of blood metals as potential upstream modifiable risk factors to prevent the development of other established risk factors as hyperhomocysteinemia.

## 1. Introduction

Homocysteine (Hc) is a sulpho-amino acid formed by interconversion of methionine and cysteine (Cys). Most of the Hc in the blood is in a protein-bound state, although some are oxidized to Hc and homocysteic acid (HCA) or forms mixed heterodimers with Cys [1,2,3]. It is present in three different forms: around 1% circulates as free thiol, 70–80% remains disulfide-bound to plasma proteins, mainly albumin, and 20–30% combines with itself to form the dimer Hc or with other thiols [4,5,6]. On the whole, the different forms of Hc in the blood are determined as “total Hc,” which in healthy humans is low, and its level is between 5.0 and 12.0 µmol/L (fasting) [4]. 

Hyperhomocysteinemia (hHc) is defined as a medical condition characterized by an abnormally high level (above 15 µmol/L) of Hc in the blood [7]. If the level is among 16–30 μmol/L, it is classified as moderate hHc, 31–100 μmol/L is considered intermediate hHc, and a value above 100 μmol/L is classified as severe hHc [4]. The biochemical change that is common to all disorders indicates that hHc is a risk factor for numerous chronic degenerative diseases, in particular, cardiovascular disease [1].

Hyperhomocysteinemia may occur from genetic defects of enzymes involved in Hc metabolism. The enzymes involved can be 5,10-methylene tetrahydrofolate reductase (MTHFR), methionine synthase, and cystathionine-β-synthase (CβS) [8]. The most frequent genetic defect is the single nucleotide polymorphisms of MTHFR that is identified worldwide, which has been associated with moderate hHc and intermediate hHc [8]. The most frequent of the genetic causes of severe hHc is supposed to be a homozygous deficiency of CβS [6]. Further rare causes of severe hHc are a homozygous deficiency of MTHFR, deficiency of methionine synthase, and impaired activity of methionine synthase, as a result of genetic disorders of vitamin B_12_ metabolism [4].

Furthermore, hHc can occur from nutritional deficiencies of folate, Vitamin B_6_, and Vitamin B_12_ [6,8]. Folate, Vitamin B_6_, and Vitamin B_12_ blood levels are inversely correlated with Hc blood concentration; consequently, nutritional deficiency leads to increase risk for hHc [6,8]. 

High Hc level increases the risk of developing heart disease, stroke, and peripheral vascular diseases, and the neurological level may be the cause of cognitive impairment [1]. Hyperhomocysteinemia may cause vascular damage by impairing vascular endothelial, smooth muscle cells function. This impairment may be brought about by the proliferation of vascular smooth muscle cells, altered elasticity of the vascular wall, the inhibition of nitric oxide synthesis, and the increase of oxidative stress [9]. Furthermore, hHc increases the generation of reactive oxygen species (ROS) and the release of arachidonic acid from platelets, inhibits glutathione (GSH) peroxidation, and it is quickly accumulated inside the cells, where it undergoes auto-oxidation and transformation into HCA. The process is accompanied by H_2_O_2_ accumulation and the appearance of HCA, which is a more damaging compound than Hc itself [8]. Endothelial cells are also sensitive to hHc, which induces oxidative damage. Various studies have demonstrated that Hc induces mitochondrial dysfunction through the regulation of oxidative stress [9,10].

Chronic exposure to heavy metals like lead (Pb), cadmium (Cd), mercury (Hg), and chromium (Cr) has been associated with a high risk of cardiovascular and nervous system disease and cancers of bladder, prostate, kidney, liver, lung, and skin [11,12,13,14,15,16,17,18].

Recent investigations suggest that toxic heavy metals may have adverse effects on these outcomes even at lower concentrations [11]. In addiction, a co-exposure of different toxic heavy metals, as usually happens in workers, could be a significant risk factor.

Moreover, recent scientific evidence suggests that exposure to toxic heavy metals may be an independent risk factor for heart disease, stroke, and peripheral vascular diseases [11]. Nevertheless, despite their well-established function as immunotoxicants and carcinogens, the correlation between toxic heavy metals exposure and risk for cardiovascular disease remains less well deepened.

Some evidence showed that exposure to these metals increased the plasma Hc level [11,12]. Damages caused by exposure to heavy metals are widespread and even were inhibited the proliferation of endothelial cells that can lead to atherosclerosis [13]

Results obtained from an experimental study illustrated that Cd and Pb, besides their direct action on blood vessel cells, could be absorbed by immune cells [19]. Studies conducted on occupational exposure to Pb revealed that the increase in blood Pb level caused an augment in systolic blood pressure, which is an additional contributor to cardiovascular involvement [16,19].

This systematic review aims to clarify the relationship between Hc blood levels and exposure to toxic heavy metals. 

## 2. Materials and Methods

This systematic review was carried out in accordance with the PRISMA statement [20].

### 2.1. Literature Search

SCOPUS, Medline (using PubMed as the search engine), Embase, and Web of Sciences databases were searched in order to recognize relevant research available until 30 October 2019, for examining the association to toxic heavy metals (Pb, Cd, Hg, and Cr) exposure with hHc as primary outcome.

MeSH term was used with the following entry terms: “Homocysteine” AND “Lead”; “Homocysteine” AND “Chromium”; “Homocysteine” AND “Cadmium”; “Homocysteine” AND “Mercury”.

A search of the research manuscript that was suitable for inclusion in this systematic review was conducted as well, and the research papers of significance therein were collected and reviewed.

### 2.2. Inclusion and Exclusion Criteria

The following inclusion criteria were adopted: (1) studies that assessed the Hc value in relationship with toxic heavy metals exposure assessment. The following exclusion criteria were applied: (1) animal studies; (2) scientific article that was not published in English language; and (3) review or conference abstracts or letters to the editor.

For duplicate studies, the only article with further detailed information was included.

### 2.3. Quality Assessment and Data Extraction

Two reviewers (CL and VR) retrieved articles independently. The title, abstract, and full text of each potentially pertinent study was reviewed. Any divergence on the eligibility of the studies was determined throughout debate or by consulting an additional reviewer (EC). The following information was extracted from all qualified papers: authors, year of publication, nationality of subjects, study characteristics.

## 3. Results

### 3.1. Characteristics of Eligible Studies

**Figure 1:**
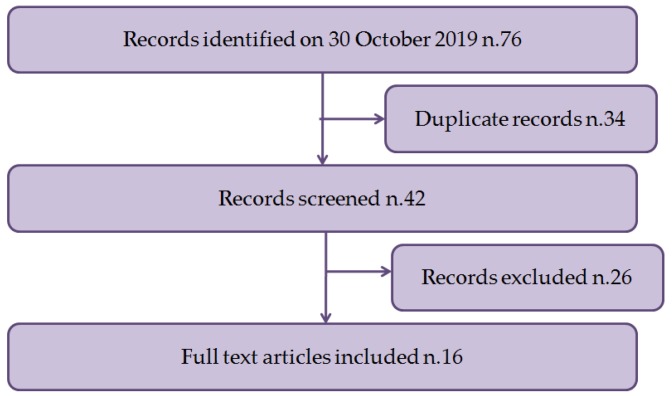
Flow diagram illustrating included and excluded studies in this systematic review.

After a free search for scientific literature by reviewers, a total of 42 documents were collected. Twelve were ruled out a subsequent review of title and abstract, and 26 studies were disqualified after review of the manuscript. In conclusion, 16 studies satisfied the inclusion criteria and were included in the systematic review [6,21,22,23,24,25,26,27,28,29,30,31,32]. A flowchart depicting the choice of studies is revealed in Figure 1. 

A summary of the details of the included research papers is reported in Table 1.

**Figure 2:**
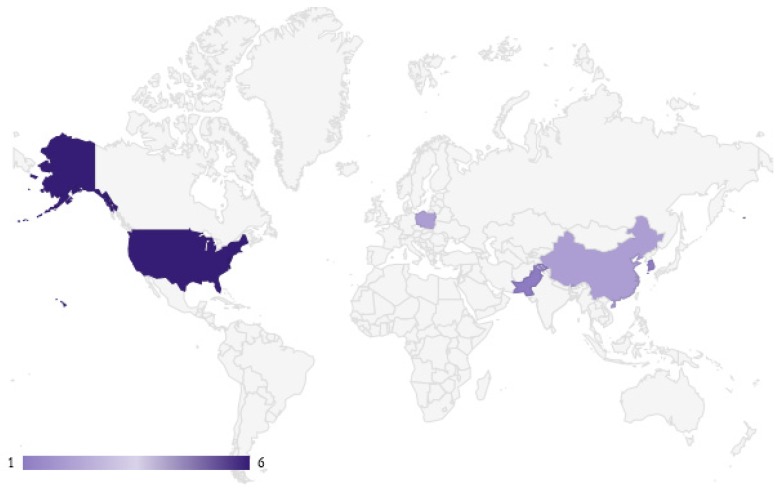
Graphical map of the geographical distribution of selected studies.

Six studies were carried out in the USA, two in China and Poland, respectively, and one in each of these countries: South Korea, Pakistan, Vietnam, and Singapore (see Figure 2).

### 3.2. Lead (Pb)

A significant number of studies (11; 69%) of the present review focus on Pb. Eight studies were carried out among the general population, while three investigations analyzed the Hc value in correlation with occupational exposure to Pb.

The range of Pb blood levels in the general population was from 0.93 (0.77–1.05) to 35.91 (23.45–48.77) µg/dL, while the Hc values ranged from 9.44 (2.0–92.6) to 14.8 ± 9.4 µmol/L. On the other hand, the Pb range of levels in occupational exposure was from 22.7 (2.0–66.9) to 43.8 ± 7.4 µg/dL, and the Hc range was from 7.8 ± 3.3 to 16.8 ± 5.24 µmol/L.

Pollak and colleagues [21], in an investigation carried out among women in reproductive age, demonstrated that blood Pb levels were correlated with increases in Hc among women consuming lower levels of essential Vitamin B_12_ and folate. This was similar to Lee et al. [27], who observed stronger associations between Pb and Hc among adult people with lesser serum values of Vitamin B_6_ and folate. In a longitudinal study, Bakulski and colleagues [25] observed older men and described that Pb and Hc were more strongly correlated among men with a lower concentration of Vitamin B_12_ and folate. Similar results were reported by Yakub and Iqbal [28] in a cross-sectional survey carried out in Pakistan. Krieg et al. [29] described that serum Hc concentration statistically significant (*p* < 0.05) increased as the blood Pb concentration statistically significant (*p* < 0.05) increased, also statistically significant (*p* < 0.05) decreased, and the serum folate and Vitamin B_12_ increased. No relationships with neurobehavioral test performance and Pb and Hc concentrations were found by Krieg and colleagues [29].

Kim et al. [22], in a population-based cohort, found an association between Pb and Hc concentrations in people living in South Korea. Furthermore, Kim reported, in the framework of genotypes for the target gene single-nucleotide polymorphisms analysis, that people with variant alleles in the transferrin gene showed a positive correlation with Pb and Hc increased levels. No association with haemochromatosis protein, betaine-homocysteine S-methyltransferase, methionine synthase (MTR), and MTHFR gene alleles was found [22].

Cai et al. [19], in an urban population living in China, revealed that blood Pb level was positively associated with Hc level. Moreover, the results underlined that Hc level was higher in the group with the highest Pb level. 

Shafer [30], in a large population-based study conducted in the USA, assessed that the relations of blood Pb with Hc levels did not differ in subgroups distinguished by age, sex, or race/ethnicity. 

In studies describing workers exposed to Pb [9,24,26], the levels of Hc were slightly higher than in the general population and confirmed that Pb blood levels were associated with high Hc levels. Kasperczyk et al. [24] reported that Pb decreases levels of GSH and protein thiol groups. Pb-induced oxidative stress contributes to the increase in protein carbonyl groups. Aside from this, Pb poisoning seems to be associated with hHc, which may promote the development of atherosclerosis. Kasperczyk et al. [24], moreover, promoted the N-Acetylcysteine (NAC) administration in Pb-exposed workers and observed that treatment with NAC normalized the level of Hc and decreased oxidative stress, as measured by the protein carbonyl content [24].

### 3.3. Mercury (Hg)

Pollack and colleagues reported that Hg blood concentration was associated with decreased Hc levels among women with serum folate < 24.49 ng/mL [21]. Another study [31] observed an inverse association between Hc and Hg. Children with a high level of blood Hg showed a low concentration of Hc. To date, no study relating Hc and Hg was carried out on workers.

### 3.4. Chromium (Cr)

Wang [32] has deepened a study on folate deficiency and selected tumor-marker concentrations in workers with long-term exposure to hexavalent chromium, in a case-control study. The plasma Hc level of workers was significant higher than in control (*p* < 0.05). Moreover, Hc concentrations were significant higher in smokers (*p* < 0.05). However, the study does not investigate the health status of workers.

### 3.5. Cadmium (Cd)

Pollack et al. [21] reported no association between Cd and Hc blood concentrations in women of reproductive age who regularly took folate and Vitamin B_12_. On the contrary, Cai et al. [23], in a study of the general population living in China, described that Hc level in the group with a high level of Cd was higher than that in the group with the lowest Cd level. Even in the case of Cd, no research has been done among workers.

## 4. Discussion

### 4.1. Lead (Pb)

Several epidemiologic and experimental studies have underlined that exposure to Pb increases the risk of hypertension [33]. Other research has demonstrated strong evidence that Pb exposure is a risk factor for ischemic heart disease [34] and overall cardiovascular morbidity and mortality [33]. 

The deleterious effect of Pb depends on the exposure period, received dose, route of absorption (respiratory system, digestive system and/or skin), presence of other xenobiotics, age, sex, and genetic factors [35]. After absorption by inhalation, Pb penetrates directly into the circulatory system [13]. In contrast, after entering through the gastrointestinal tract, Pb is absorbed into the stomach and small intestine and transported first into the liver and then into the general circulation [36].

However, regardless of the route of entry into the organism, its distribution and accumulation always show a similar pattern [24]. Pb is not easily eliminated from the body and can reside in the body (liver, spleen, kidneys, and especially bones) for years [9].

All studies of this review support that Pb concentrations are correlated with high levels of Hc [9,21,22,23,24,25,26,27,28,29,30].

Mechanistically, Pb may affect Hc concentrations via various pathways. Pb has been associated with oxidative stress and dysregulations in the nitric oxide system and inflammation processes [37,38]. Pb can also interact with sulfhydryl groups, consequently with Hc being a sulfur-containing amino acid [30,39]. Pb exposure could adduce to copper (Cu) deficiency [40]. Diets with high Cu were associated with slight declines in folate and Hc concentrations [41], which may mean that diets low in Cu could also affect Hc concentrations.

Hc is correlated with the development of atherosclerosis; nevertheless, the mechanism by which Hc induces atherosclerotic lesions is not well-known [42,43,44]. Hc impairs the function of vascular endothelial smooth muscle cells, induces their proliferation, alters the elasticity of the vascular wall, and increases oxidative stress through the production of reactive oxygen species (ROS) [42,43,44].

At the same time, in numerous investigations in humans and animals exposed to Pb, the increase in ROS production was observed [42,43,44,45,46,47,48,49].

ROS, including superoxide anion, hydroxyl radical, and lipid radicals, provoke the chemical alteration of macromolecules, which results in disturbing the organization of biological cell membranes in several organs within the organism and the injury of the cell’s role [50,51]. ROS has a key function in the pathogenesis of cardiovascular disease, where there is distressed physiological stability between ROS cell production and antioxidative activity in the organism [49,50,51]. Other significant factors include dysregulation of calcium channels [52], altered activity of proteins and tyrosine kinases [53], changed transcription factors activity [54,55,56,57] and NO synthase activity [58,59], and an increase in levels of inflammatory markers and functional changes in immunological molecules, like Hc [60,61,62].

Kim and colleagues [22] underlined that blood Pb concentrations were positively associated with high Hc plasma levels and variant alleles in transferrin gene. This suggests that iron (Fe) distribution may influence the effect of lead toxicity on Hc concentration by affecting Hc metabolism. Genetic polymorphisms in the transferring gene can induce changes in Fe distribution, haeme synthesis, and activity of CβS, a haeme-containing enzyme that metabolites Hc into CβS in a trans-sulfuration pathway, resulting in a gradient in Hc concentrations [63,64,65]. While Pb leads to decreases haeme and haemoglobin synthesis and CβS activity, synergistic increases associated with Pb exposure may not occur at high concentrations of Hc, possibly because of the low CβS activity [27].

Bakulski [25] and Lee [27] observed a statistically adverse effect between blood Pb level and the micronutrients folate, Vitamin B_6_, and Vitamin B_12_, which are involved in transmethylation or trans-sulfuration metabolism on Hc concentrations. As described, blood Pb may inhibit Hc metabolism [27]. There is a hypothesis that Pb might directly inactivate the enzymes implicated in methionine metabolism or may adhere competitively to the active site for Hc or Hc itself. Pb can also biologically interact with folate, Vitamin B_6_, and Vitamin B_12_ [27].

Kasperczyk [24] reported the excellent effect of NAC administration on Hc concentration concerning oxidative damage to protein and levels of Fe and Fe related protein in a group of workers exposed to Pb. The treatment with NAC confirms that the anti-inflammatory properties have not been studied enough [66,67]. 

It might be essential to maintain adequate levels of micronutrients to prevent potential Pb exposure from harming the metabolism of Hc. In the case of chronic exposure to Pb, NAC administration could be included in protocols for the treatment of Pb toxicity based on NAC anti-inflammatory properties.

### 4.2. Mercury (Hg)

Low Hc concentrations were associated with Hg serum concentrations in adults [21]; comparable outcomes were observed in children, but only for boys [31].

The mode of action by which Hg is associated with low values of Hc in the presence of decreased concentrations of serum folate could be due to an increased requirement for GSH, which induces Hc to be implicated in GSH production instead of methylating methionine [68]. This development would lead to lower Hc values. Hg has an affinity to Selenium (Se), an essential nutrient which may play a function in its slightly negative correlation with Hc. Studies underlined that plasma Hc was significantly reduced in Se-deprived mammifers [69].

As of worry concerning the toxicity of Hg, it is essential to recognize the forms of Hg which have the characteristic of being transported to target organs and cells. Mercuric ions have a strong affinity intended for reduced sulfur atoms [70]. Therefore, mercuric ions inside biological systems are transformed for the most part to conjugates of one or more sulfur (thiol)-containing biomolecules, such as GSH, Cys, and Hc. Some of these Hg conjugates are comparable structurally to some endogenous amino acids [71,72,73]. For instance, the structure of the Cys *S*-conjugate of methylmercury (CH_3_Hg-*S*-Cys) is analogous with the structure of methionine. CH_3_Hg-*S*-Cys can be produced by the direct reaction of Cys with methylmercury. Instead, methylmercury may act in response with GSH to form a GSH *S*-conjugate (CH_3_Hg-*S*-G), which can then be catabolized to CH_3_Hg-*S*-Cys by the sequential activities of γ-glutamyltransferase and cysteinylglycinase/aminopeptidase M located in epithelial cell [71,73]. Because of the attendance of a large hydrophobic side group and structural connection to methionine, CH_3_Hg-*S*-Cys may be transported addicted to cells via the amino acid transporter, system L [73]. This transporter has a similarity with large hydrophobic amino acids, as well as L-methionine [73].

The connection between CH_3_Hg-*S*-Cys and methionine has been highlighted [74,75,76,77] to be a case of “molecular mimicry”, whereby CH_3_Hg-*S*-Cys is a mimic of methionine at the site of the system L transporter. Molecular mimicry has been classified as the occurrence whereby one molecule or compound can actively resemble the structure or function of another endogenous molecule or compound [71,75].

The mechanism of action by which Hg is correlated with low concentrations of Hc in lower concentrations of serum folate can be amplified when requiring for GSH, which induces Hc to be implicated in GSH production instead of methylating methionine. This procedure causes lower Hc concentrations.

### 4.3. Chromium (Cr)

Some experimental studies explain that Vitamin B_12_ and folate participate in maintaining the stability of the genome and that an insufficiency of them can be correlated with chromosomal damage [78]. Further studies have suggested that Cr can accumulate in the kidney tissues, causing both tubular and glomerular renal impairment. The kidney was considered the target organ of Cr toxicity [79]. Previous studies have shown the importance of kidney for the metabolism of Vitamin B_12_ and folate; the alteration of vitamins’ metabolism may be a consequence of kidney disease [79]. Vitamin B_12_ and folate deficiency can cause elevated blood concentrations of Hc, which is associated with an increased risk of developing cardiovascular diseases [80]. In addition to Vitamin B_12_ and folate, the kidney also metabolizes Hc; elevated blood levels of Hc have been found in patients with kidney disease [80,81]. Although the pathogenesis of hHc in renal disease is still unknown, both in vivo and in vitro studies have suggested that the kidney plays a fundamental role in Hc metabolism [81,82]. Data from previous studies show that high blood Hc values in patients with renal disease are related to the reduction of Hc clearance from the body due to metabolic accumulation [83,84]. Although the adverse effects of Cr on DNA and the damage caused are well-known, there are no published data on the metabolic changes of folates, Vitamin B_12_, and Hc in chronically exposed workers. The relationship between Vitamin B_12_ levels, folates, Hc, and Cr exposure in workers of chromate manufactured articles has not been studied, and information on the effect of renal impairment on Vitamin B_12_, folates, and plasma levels of total Hc is lacking [79].

### 4.4. Cadmium (Cd)

Cd can cause damage to DNA, trigger oxidative stress, disrupt protein function, and cause several illnesses [85]. Exposure to heavy metals is linked to high incidence rates of cardiovascular diseases as well [86]. Epidemiological studies showed that exposure to Cd increased the risk of cardiovascular diseases, and a low level of Cd exposure is one of the decisive factors in cardiovascular mortality [19]. Cd and Pb can be considered the main heavy metal pollutants affecting human health [87,88]. Elevated Hc level in the human body affects the body in different ways; it can accelerate oxidation, aging, and damage to arteries, weakening the immune system and speeding up brain aging. Studies have shown that increased Hc levels can lead to a proliferation of vascular smooth muscle cells and damage the cardiovascular system. High blood lead and Cd levels, along with high Hc levels, can negatively affect both the cardiovascular and nervous system [19].

## 5. Conclusions

Hc is a sulfur amino acid whose metabolism is activated in two pathways: remethylation to methionine, which requires folate and Vitamin B_12_, and trans-sulfuration to cystathionine, which needs pyridoxal-5’-phosphate. The two pathways are coordinated by S-adenosylmethionine, which operates as an allosteric inhibitor of the methylenetetrahydrofolate reductase response and as an activator of CβS. 

The result of this systematic review indicates that exposure to Pb, Cr, Cd, and Hg is connected with non-physiological serum concentrations of Hc, Vitamin B_12_, and folate. 

These findings reinforce the (often under-recognized) importance of the involvement of exposure to heavy metals in Hc metabolism. This supports the role of blood metals as potential upstream modifiable risk factors to prevent the development of other established risk factors for hHc.

Furthermore, it is recommended to determinate Hc levels in subjects exposed to heavy metals, to approach the concept of *precision medicine*, an emerging approach for disease treatment and prevention based on subject characteristics such as genetics and environmental/occupational exposure.

## Figures and Tables

**Table 1:** Characteristics of eligible studies.

Reference	Population	Exposure	*n* *	Age	Country	Intervention/Outcome	Toxic Heavy Metals Mean Level (Pb = µg/dL; Hg, Cr, Cd = µg/L)	Hc mean Level (µmol/L)
Pollack et al., 2017 [21]	Women of reproductive age	Pb Non-occupational exposure	259	27.4 ± 8.2	USA	Toxic heavy metals value; dietary intake of folate and vitamins B	0.93 (0.77–1.05)	10.1 (2.0–12.9)
Kim et al., 2017 [22]	Population-based cohort	Pb Non-occupational exposure	386	55	South Korea	Pb value; gene polymorphisms and Hc interaction	4.4 ± 1.9	11.1 ± 1.4
Cai et al., 2017 [23]	Urban populations	Pb Non-occupational exposure	159	n.d.	China	Pb value; Male/Female Smoking/non-smoking Drinking/non-drinking	35.91 (23.45–48.77) M 27.10 (21.46–32.76) F	12.65 ± 4.73 M 11.96 ± 3.88 F
Kasperczyk et al., 2016 [24]	Lead-exposed workers	Pb Occupational exposure	171	42.5 ± 8.66	Poland	Pb value; influence of *N*-acetylcysteine administration on Hc level	43.8 ± 7.4	15.6 ± 5.38 ^§^
Bakulski et al., 2014 [25]	Men with ≤55 yrs old	Pb Non-occupational exposure	1.056	69 ± 7.4	USA	Pb value; dietary intake of folate and vitamins B	4.9 ± 2.7	10.1
Kasperczyk et al., 2013 [26]	Lead-exposed workers	Pb Occupational exposure	183	42.2 ± 8.93	Poland	Exposure to Pb and effects on biothiols metabolism	Low-Pb 38.4 ± 5.44 High-Pb 49.2 ± 4.01	Low-Pb 14.8 ± 4.55 ^§^ High-Pb 16.8 ± 5.24 ^§^
Lee et al., 2012 [27]	General populations	Pb Non-occupational exposure	4089	50.4 ± 19.3	USA	Association between Hc, Pb, and micronutrients	2.1 ± 1.8	Positive trend with Pb levels
Yakub et al., 2010 [28]	General populations	Pb Non-occupational exposure	872	32.51 ± 10.71	Pakistan	Pb blood level and risk of hHc	11.65 ± 5.5	14.8 ± 9.4 ^§^
Krieg et al., 2009 [29]	General populations	Pb Non-occupational exposure	2823	Range: 20–59 years	USA	Pb blood and Hc levels and neurobehavioral performance	2.88 (0.7–28.1)	9.44 (2.0–92.6)
Chia et al., 2007 [9]	Lead-exposed workers	Pb Occupational exposure	449	38.7 ± 10.7	Vietnam and Singapore	Pb blood level and risk of hHc	22.7 (2.0–66.9)	7.8 ± 3.3
Schafaer et al., 2005 [30]	General populations	Pb Non-occupational exposure	1037	59.3 ± 5.9	USA	Pb blood level and risk of hHc	3.5 (0.1–27.3)	10.0 ± 4.1
Pollack et al., 2017 [21]	Women of reproductive age	Hg Non-occupational exposure	259	27.4 ± 8.2	USA	Toxic heavy metals value; dietary intake of folate and vitamins B	1.05 (0.93–1.18)	10.1(2.0–12.9)
Gallagher et al., 2011 [31]	Children	Hg Non-occupational exposure	1005	Range: 3–5 years	USA	Total blood Hg, Hc, methylmalonic acid, and folate	Boys: 0.50 (mean) Girls: 0.79 (mean)	Boys: 4.53 (mean) Girls: 4.39 (mean)
Wang et al., 2014 [32]	Workers	CrVI occupational exposure	115	37.96 (5.93)	China	Folate deficiency and tumor marker concentrations	12.45 (20.23)	Higher in CrVI exposed workers
Pollack et al., 2017 [21]	Women of reproductive age	Cd Non-occupational exposure	259	27.4 ± 8.2	USA	Toxic heavy metals value; dietary intake of folate and vitamins B	0.29 (0.26–0.31)	10.1(2.0–12.9)
Cai et al., 2017 [23]	Urban populations	Cd Non-occupational exposure	159	n.d.	China	Pb and Cd value; Male/Female Smoking/non-smoking Drinking/non-drinking	1.11 (0.55–2.75) M 0.72 (0.29–1.33) F	12.65 ± 4.73 M 11.96 ± 3.88 F

* Number of subjects studied; n.d. = not declared; ^§^ moderate hHc.
